# Inverse Co‐Design of Mechanical And Sensory Properties in Soft Lattice Foams for Multifunctional Wearables

**DOI:** 10.1002/advs.202507102

**Published:** 2025-09-23

**Authors:** Qinghua Guan, Benhui Dai, Harshal Sonar, Josie Hughes

**Affiliations:** ^1^ CREATE Lab EPFL Lausanne 1015 Switzerland; ^2^ Bearmind SA EPFL Innovation Park Bat. C Lausanne 1015 Switzerland

**Keywords:** inverse design, lattice foams, neural network, soft sensors, wearable devices

## Abstract

Lattice structures exhibit a variety of topological geometries, which endow them with diverse mechanical properties and high design flexibility, and enable them to be integrated with sensing mechanisms to collect environmental or intrinsic information. However, the traditionally empirical and separated design leads to difficulties in the integration of multiple functions and limits the exploration of the extensive properties of lattice foams. In this work, a design methodology is proposed for the inverse co‐design of the mechanical and sensory properties of lattice foams by training an inverse‐design neural network and optimizing the layer laminating permutations. Physics‐informed numerical models and hybridization principles are employed to generate training data for the inverse mechanical design of hybrid foams. Subsequently, the mechano‐electrical relationships are defined and utilized to predict and optimize the sensory behaviors of the hybrid foams. As a proof‐of‐concept example, a smart knee pad is developed to meet a specified target protective function and optimized sensory behavior, demonstrating not only its inverse‐designed mechanical response but also its sensing capability from light interaction forces with users to heavy impact forces from collisions. This concept allows for more data‐driven analytical approaches to satisfy the various needs for multi‐functional wearable devices.

## Graphical abstract

An inverse co‐design framework integrates neural networks and physics‐based models to engineer soft lattice foams with independently tunable mechanical and sensory properties. By layering architected foams and optimizing electrode placement, this method enables multifunctional wearable sensors. A smart knee pad showcases targeted protection and capacitive sensing, revealing novel strategies for personalized wearable devices.

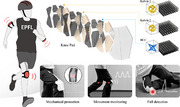

## 1 Introduction

With advances in 3D printing technologies, the fabrication of open lattice foam structures with complex and spatially varying geometries is becoming possible.^[^ ^1^, ^2^, ^3^, ^4^ ^]^ Such foam structures have increasing applications in soft robotics^[^ ^5^, ^6^ ^]^ and wearable applications^[^ ^7^ ^]^ due to their advantages over traditional foam padding, including enhanced energy absorption, customization, and improved breathability. Another emerging advantage of these structures is the capacity to functionalize and sensorize them so they can sense deformation and compression in addition to having advantageous mechanical properties.^[^ ^8^ ^]^ Sensing can be incorporated through a number of techniques, including fluidic innervation,^[^ ^9^ ^]^ incorporating piezoresistive materials,^[^ ^10^ ^]^ or capacitative sensing where the foam is used as a dielectric.^[^ ^11^ ^]^ Capacitative sensing is particularly interesting as the sensing modality is coupled with the mechanical properties,^[^ ^12^, ^13^ ^]^ and unlike many other sensing modalities, the output is unaffected by changes in ambient temperature or pressure.^[^ ^14^, ^15^ ^]^ However, to fully leverage the advantages of customizable lattice foams, designs are required that enable a large range of possible mechanical and sensory properties. Stemming from this, we then require methods which can solve the inverse 'co‐design' problem, i.e., identify the foam geometry to achieve the required mechanical and sensor properties.

Lattice structures are typically formed from geometric repeats of the same lattice unit cell^[^ ^16^ ^]^ such as body centered cubic (BCC),^[^ ^17^ ^]^ Kelvin^[^ ^18^ ^]^ or octahedron^[^ ^19^ ^]^ This limitation constrains the range of possible mechanical properties, namely the stress strain behavior, of the lattice foam. There has been some limited exploration of more complex geometries in architected materials. In one example, the regulation of the thickness or topology of the structures is used to create spatially varying mechanical properties, which expands the range of possible mechanical behaviors.^[^ ^20^, ^21^, ^22^ ^]^ A second challenge is the geometrical programming of the mechanical and sensing properties of the lattice structure, and how to solve the inverse design problem of designing a lattice geometry to achieve a desired mechanical and sensory performance. One typical approach is to use finite element method (FEM) to predict the behaviours of different foams.^[^ ^6^, ^23^ ^]^ FEM has used alongside optimization algorithms for rigid lattice structures, for example with a goal to optimize properties such as stiffness, fracture toughness, impact or vibration absorption, and thermal transmission^[^ ^24^, ^25^, ^26^, ^27^, ^28^, ^29^ ^]^ Recently, inverse‐designed spinodoid metamaterials leveraged an machine‐learning‐based inverse‐design framework, trained on phase‐field and FEM data to yield nonperiodic cellular architectures with tunable anisotropic stiffness and enhanced energy absorption.^[^ ^30^ ^]^ However, soft lattice structures^[^ ^31^, ^32^, ^33^, ^34^ ^]^ exhibit complex mechanical behaviors due to material and geometry non‐linearity (e.g., material super‐elasticity and beam buckling) when undergoing large deformation^[^ ^9^, ^35^ ^]^ rendering FEM insufficient or highly compute intensive. Additionally, co‐design requires simulation of mechanical and sensory properties which further complicates the analysis, rendering standard FEM techniques insufficient. Consequently, alternative approaches are necessary for the inverse design of soft, sensorized lattice foams.^[^ ^22^ ^]^

**Figure 1:**
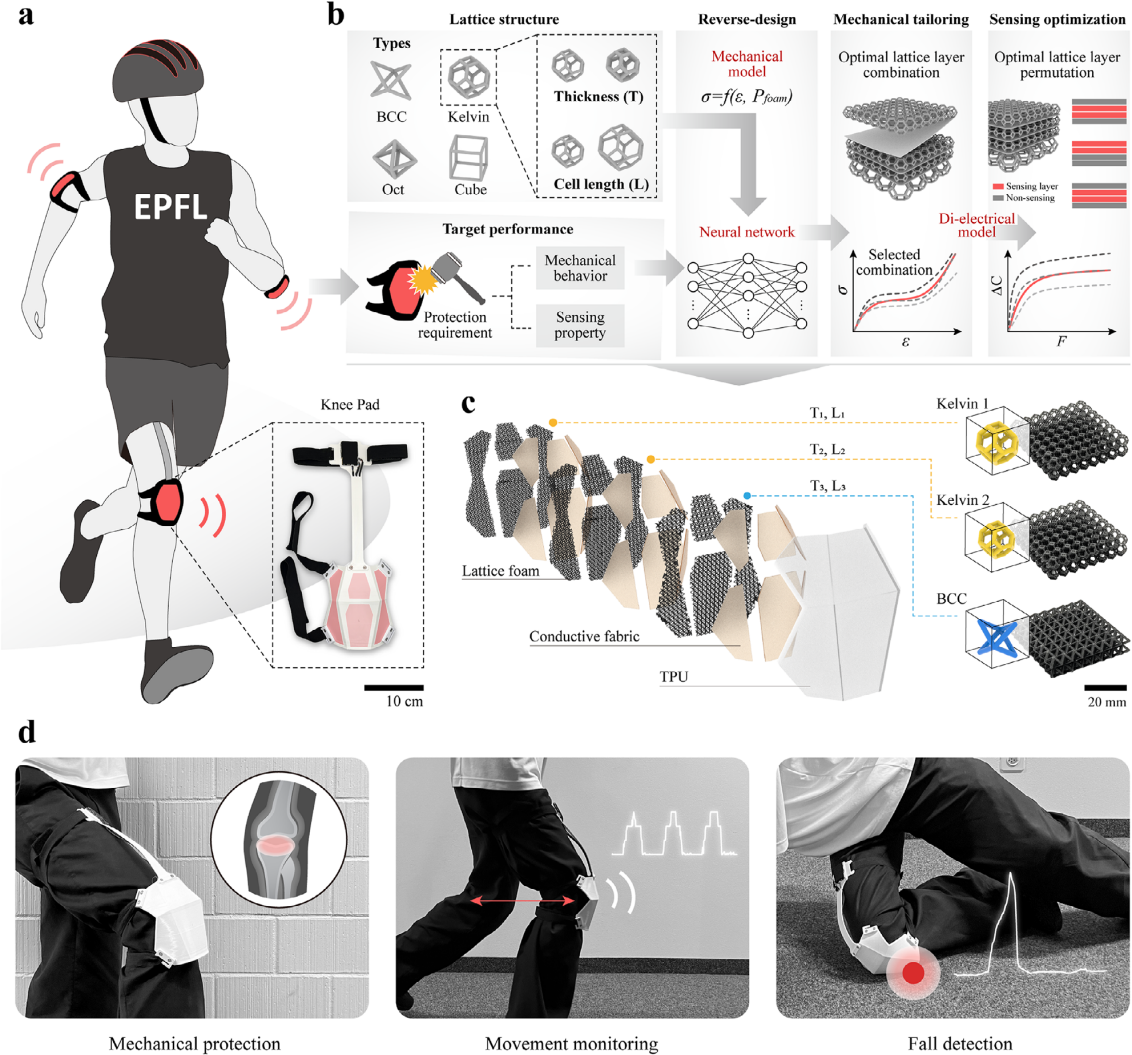
Soft lattice foams (SLFs) with tailored protection and sensing capabilities for wearable gears. a) Wearable protective gear. b) Methodology for the design and fabrication of SLF. c) The design of the knee pad utilizing SLF. d) Applications of SLF.

Leveraging recent advancements in 3D printing and machine learning technology we introduce a novel design space and method for the mechanical and sensory programming of soft lattice foams (SLFs),^[^ ^16^, ^36^, ^37^ ^]^ our approach is summarized in **Figure** **1**b. We propose forming SLFs by laminating layers of different lattice structure where their cell length and thickness can also be varied. By incorporating soft conductive layers within the SLFs, capacitative sensing is introduced to these hybrid foams. Importantly, by changing the layer ordering, the sensory properties can be varied while the mechanical properties remain constant, providing the capacity for some independent optimization of the mechanical and sensory performance. Thus, this approach not only enables a wider range of tailored mechanical properties but also integrates sensing without affecting mechanical performance whilst still allowing for independent optimization of sensory and mechanical response. By developing numerical models for different cell types that are physically grounded and correlated with known deformation mechanisms, we can predict the behaviors of soft foam layers. These numerical models are used to develop a data‐driven approach for inverse design of mechanical and sensory properties. To do so we propose a pipeline using a neural network to perform the inverse design of multilayer hybrid lattice foam structures for given mechanical behaviors, followed by a secondary step to optimize the placement of electrodes for the required sensory capabilities. This approach is necessary to fully exploits the wide possible range of mechanical behaviors enabled by the hybrid multi‐layer structure of the SLFs, and to exploit the layered structure for optimization of sensor and mechanical properties.

We demonstrate this approach on two classic lattice structures, the BCC and Kelvin lattices, where we experimentally investigated their change in mechanical properties with varying thicknesses and cell lengths to develop a physics‐informed numerical model for predicting their behaviors based on geometrical parameters. Our layered hybridization approach is used to generate a hybrid foam database for training a neural network which can then be used to perform an inverse design, generating the hybrid foam structure to achieve the target mechanical behavior. The mechano‐electrical relationship are demonstrated using BCC foam sensors with embedded soft fabric layers that enable the sensory behavior of the soft lattice foam.

To demonstrate the capabilities of the foams and the inverse design methods, we consider the application scenario of sensorized protective foams, which offer mechanical protection and also measure impact and contact forces. Target mechanical and sensory behaviors are choose to reflect the needs of different protective foams, and their design is determined using our inverse design method to closely meet the proposed design objectives. Finally, the sensing response is optimized by selecting the preferred combination of electrode and lattice layers, to obtain the most suitable sensing behavior. Using, this we demonstrate the co‐optimization of both the mechanical properties and sensing capabilities of our smart foam. We showcase this through a knee pad example where the SLFs is optimized for both protection and sensing. The knee pad can not only mitigate and detect heavy external impacts but also perceive light interaction forces with the wearer.

## 2 Results

The combination of advanced lattice structures and material customization opens new possibilities for achieving multifunctional properties in foam designs. By stereolithography (SLA) 3D printing of polyurethane (PU), we explore how mechanical and sensory capabilities can be optimized within hybrid lattice foams. In this section, we first present the range of mechanical properties obtained through different lattice geometries and hybrid configurations. These foams were fabricated and characterized to develop physics‐based predictive models for the inverse design of mechanical behaviors. We demonstrate how closely the inverse design process can match target mechanical responses. Following this, we highlight the capacitive sensing capabilities of the foams, showing how these sensory behaviors can be fine‐tuned to complement the desired mechanical characteristics.

### 2.1 Modeling and Characterization of Lattice foams

To optimize the mechanical performance of foam structures for different applications, it is imperative to investigate a broad range of foam geometries. The foam geometry can vary structural properties including the effective elastic modulus, yield strain, and densification strain of the lattice structure. However, experimental data collection over a wide range of foam geometries is resource‐intensive and time‐consuming. Consequently, it is crucial to develop mechanical models for mono‐/hybrid lattice foams based on experimental data, to expand the database for training a mechanical inverse design neural network. The Kelvin and BCC structures are two fundamental lattice geometries that exhibit distinct stress–strain behaviors. Their geometric parameters, such as beam thickness $T_{b}$ and cell length $L_{c}$, significantly influence their mechanical properties. By strategically varying these parameters in a layered structure to create hybrid foams, a broader range of mechanical behaviors can be achieved. In the following section, we first focus on characterization and modelling of the Kelvin and BCC foam before progressing to the hybrid foams.

#### 2.1.1 Parameterized Kelvin and BCC Foams

##### Kelvin Foams

**Figure 2:**
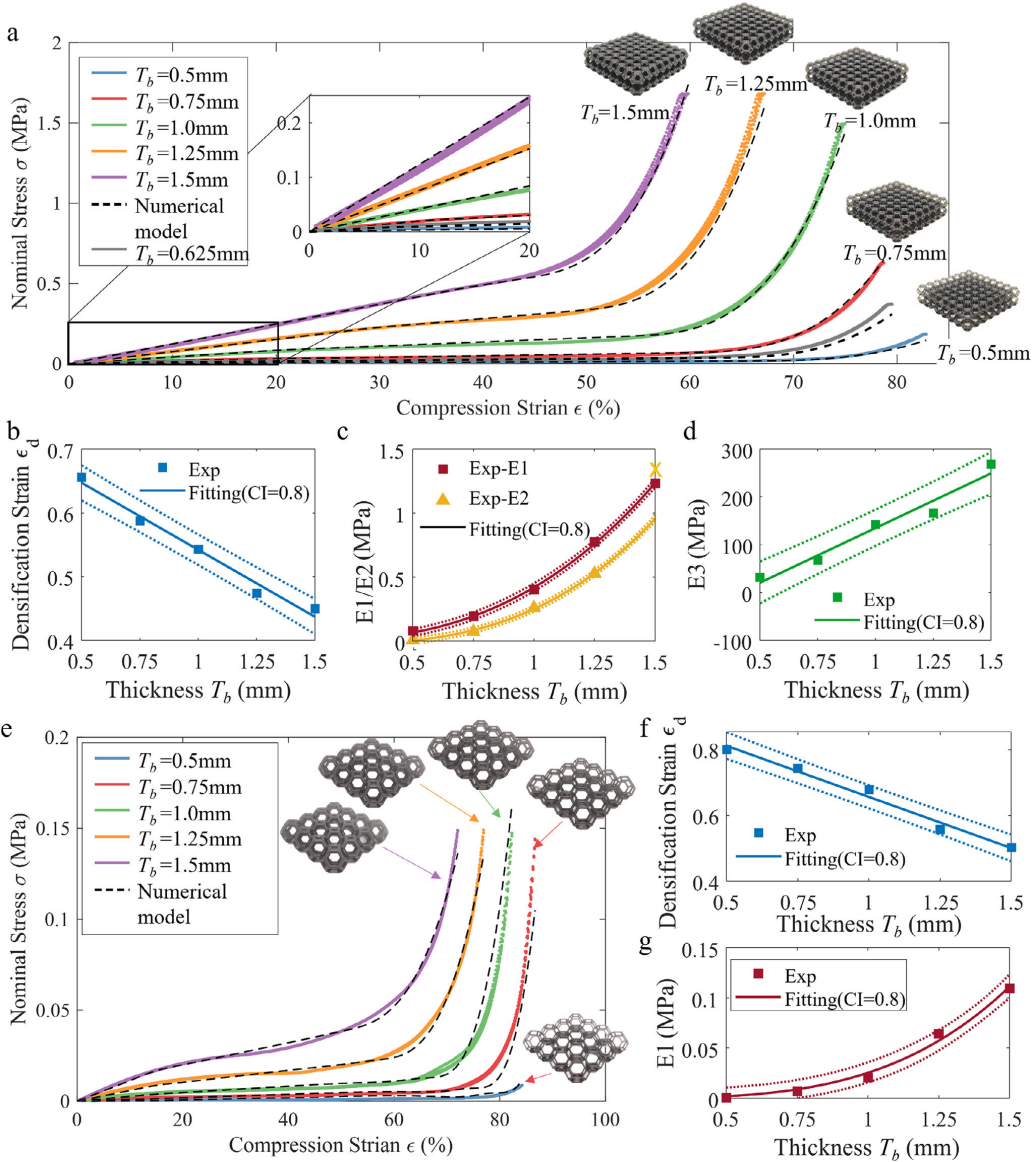
Kelvin foams with varied beam thickness and cell length. a) The experimental and modeling results of foams with cell length $L_{c}$ = 5 mm at varying beam thicknesses $T_{b}$. b‐d) The relationship between the mechanical parameters and the beam thickness $T_{b}$ of foams with $L_{c}$ = 5 mm. e) The experimental and modeling results of foams with $L_{c}$ = 10 mm at varying beam thicknesses $T_{b}$. f,g) The relationship between the mechanical parameters and the beam thickness $T_{b}$ of foams with $L_{c}$ = 10 mm.

Kelvin lattice foams were fabricated with varying beam thicknesses ($T_{b}$ = 0.5, 0.75, 1.0, 1.25, and 1.5 mm) and two cell lengths ($L_{c}$ = 5 mm and 10 mm). To investigate their mechanical behavior compression tests were performed using an Instron universal testing machine under a quasi‐static loading speed of 0.5 mms^−1^. The corresponding stress–strain responses for these geometries are presented in **Figure** **2**.

The mechanical behavior of soft Kelvin lattices is governed by a combination of stretching and bending dominated strut deformation mechanisms. According to classical lattice mechanics, the effective modulus E scales as $T_{b}^{2}$ under stretching‐dominated deformation and as $T_{b}^{4}$ under bending‐dominated conditions. Therefore, it is assumed that the scaling exponent α in the relation $E\propto T_{b}^{\alpha }$ typically ranges between 2 and 4, depending on the deformation regime and the strut slenderness ratio $\eta =L_{b}/T_{b}$.

To capture the distinct mechanical responses across deformation regimes, we propose a piecewise physics‐based empirical model. The first region is a linear elastic stage. This is followed by the second region, a lower gradient plateau in the stress–strain curve that occurs due to the buckling of the lattice beams when stability is lost under increasing compression forces. As the compression force continues to increase, the lattice beams come into contact with each other, leading to the third behavioral region, structural densification, where the nominal stress σ increases rapidly. The first and the second stage are assumed to have a linear stress–strain response, while the stress, σ, in the third region that undergoes densification is assumed to have the third‐order polynomial relationship with the strain ε. Thus, we can represent this relationship with the equation as below:

(1) $$\sigma =\left\{\begin{array}{cc} E_{1}\varepsilon & 0\leq \varepsilon \leq \varepsilon_{b}\\ E_{2}\left(\varepsilon -\varepsilon_{b}\right)+E_{1}\varepsilon_{b} & \varepsilon_{b}<\varepsilon \leq \varepsilon_{d}\\ E_{3}{\left(\varepsilon -\varepsilon_{d}\right)}^{3}+E_{2}\left(\varepsilon -\varepsilon_{b}\right)+E_{1}\varepsilon_{b} & \varepsilon >\varepsilon_{d} \end{array}\right.$$

where $\varepsilon_{b}$ and $\varepsilon_{d}$ denote the strain thresholds for buckling onset and densification, respectively. To ensure smooth transitions between these regimes, a hyperbolic tangent blending function is used (see Equation (S5), Supporting Information).

The scaling exponent α, reflects the relative contribution of stretching‐ and bending‐dominated mechanisms, and is influenced by both the deformation regime and the strut slenderness ratio. Thus, the effective moduli in each region can be modeled as functions of $T_{b}$:

(2) $$\begin{array}{cc} & E_{1}=a_{1}\cdot T_{b}^{\alpha_{1}},\;E_{2}=a_{2}\cdot T_{b}^{\alpha_{2}}+c_{2},\\ & E_{3}=a_{3}\cdot T_{b}^{3}+b_{3}\cdot T_{b}^{2}+c_{3}\cdot T_{b}+d_{3} \end{array}$$

where $\alpha_{1}$ and $\alpha_{2}$ represent the scaling exponents for the linear elastic and post‐buckling regimes, respectively. In the densification regime, due to the highly nonlinear and complex nature of foam compaction, a third‐order polynomial is employed to capture the dependence of the nonlinear nominal elasticity modulus $E_{3}$ on $T_{b}$, providing greater flexibility in modeling the observed non‐monotonic behavior. All parameters ($\alpha_{1}$, $\alpha_{2}$, $a_{1}$, $a_{2}$, $a_{3}$, $b_{3}$, $c_{2}$, $c_{3}$, and $d_{3}$) are extracted from curve fitting to experimental data (Figure 2a,e).

The scaling exponent α in the power‐law relationship $E\propto T_{b}^{\alpha }$ is not arbitrarily assigned; rather, it emerges from the intrinsic unit‐cell architecture, the deformation regime, and the slenderness ratio $\eta =L_{b}/T_{b}$ of the lattice struts. Experimental results demonstrate that α varies systematically with both deformation regime and slenderness ratio, as detailed in Table S1 (Supporting Information). For Kelvin lattices with a cell length of $L_{c}=5$ mm and beam thicknesses $T_{b}$ ranging from 0.5 to 1.5 mm (η=3.54 to 1.18), the pre‐buckling modulus scales as $E_{1}\propto T_{b}^{2.66}$, indicating deformation mainly dominated by axial stretching/compression. In contrast, the post‐buckling modulus follows $E_{2}\propto T_{b}^{3.10}$, reflecting an increasing contribution from bending. When the cell length is increased to $L_{c}=10$ mm (corresponding to η=7.07 to 2.36), the foam structures become significantly softer (Figure 2e). Consequently, the scaling exponents increase to $E_{1}\propto T_{b}^{3.63}$ and $E_{2}\propto T_{b}^{3.87}$, indicating a pronounced shift toward bending‐dominated mechanical response.

In addition, the densification strain $\varepsilon_{d}$ is significantly influenced by beam thickness, exhibiting a linear dependence across both cell lengths, as illustrated in Figure 2b,f. To further validate the model, a lattice foam with an intermediate beam thickness of 0.625 mm was fabricated and tested. The resulting mechanical response closely matches the model predictions, as shown by the grey curve in Figure 2a.

##### BCC Foams

**Figure 3:**
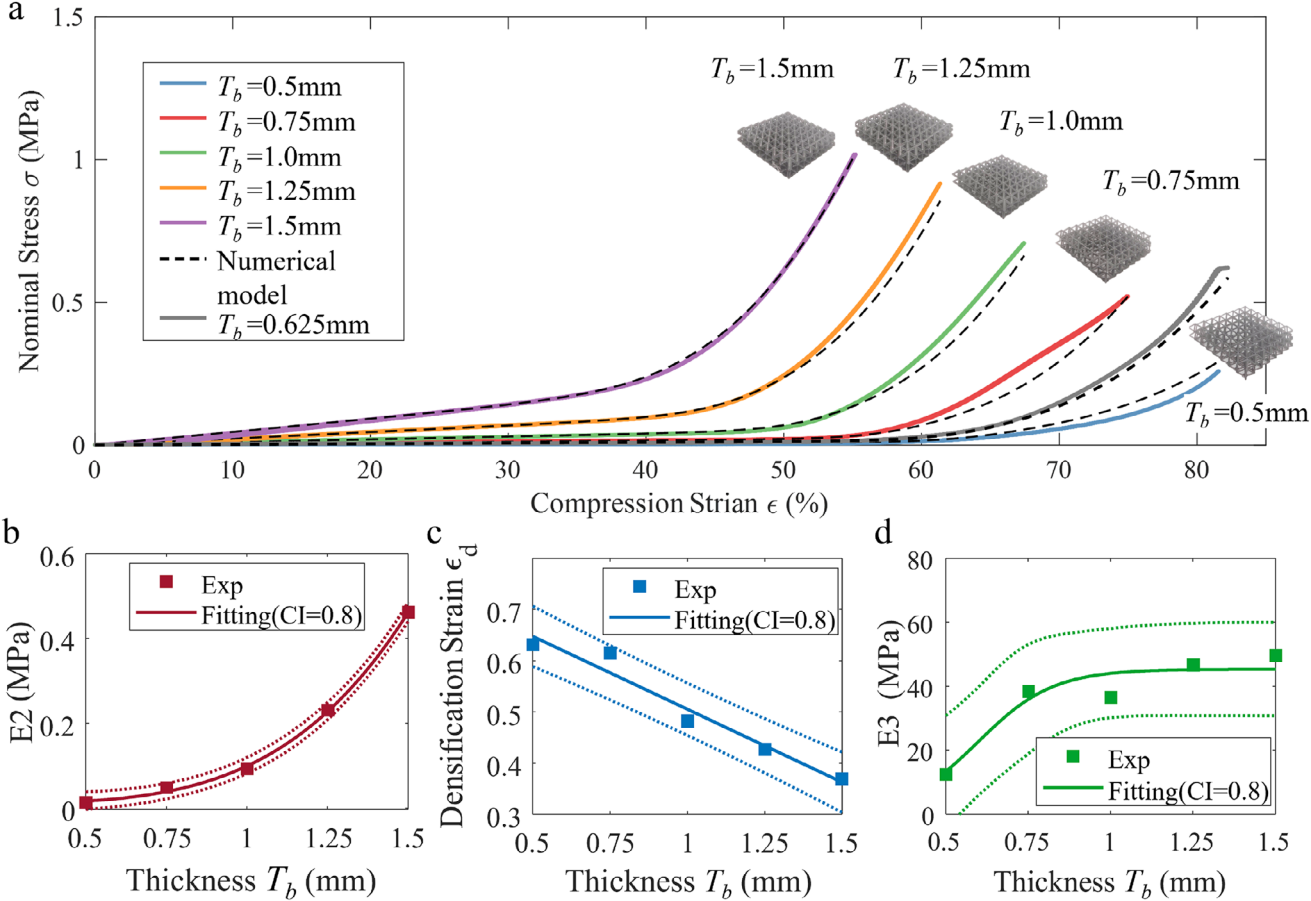
Mechanical behaviors of BCC foams with varying beam thicknesses $T_{b}$. a) The experimental and modeling results of BCC foams with cell length $L_{c}$ = 5 mm and different beam thicknesses $T_{b}$. b‐d) The relationship between the mechanical parameters and the beam thickness $T_{b}$ of BCC foams with $L_{c}$ = 5 mm.

The BCC foams are fabricated with varying beam thicknesses ranging from 0.5 to 1.5 mm, similar to Kelvin foams, and the same characterization is performed (**Figure** **3**a). Due to fabrication constraints, only a cell length of 5 mm is considered as a 10 mm cell length in the BCC lattice structure results in an excessive overhang distance, which is infeasible for soft resin printing. Additionally, Kelvin structures with a 10 mm cell length already offer adequate range of mechanical properties.

Due to the lattice topology, the beams of the BCC structure have a longer span such that they buckle at lower forces, making their first linear stage negligible. Thus, the stress–strain response of BCC lattice foam under compression can be divided into two stages, as shown in Figure 3a. In the first stage, its behavior is dominated by buckling, representing a linear stage with the nominal elasticity modulus $E_{2}$. Then, as the compressive force increases, the lattice beams come into contact with each other, rapidly increasing the stress σ. In this stage, stress σ is also assumed to have a third‐order polynomial relationship with strain ε. Furthermore, varying beam thickness results in significantly different mechanical performances, which can be accounted for by a deformation strain $\sigma_{d}$ and the nonlinear effective elasticity modulus $E_{3}$. Therefore, the mechanical behaviors of BCC foams with variable thickness can be described by a simplification of Equation (1):

(3) $$\sigma =\left\{\begin{array}{cc} E_{2}\varepsilon & \varepsilon \leq \varepsilon_{d}\\ E_{3}{\left(\varepsilon -\varepsilon_{d}\right)}^{3}+E_{2} & \varepsilon >\varepsilon_{d} \end{array}\right.$$

With this proposed analytical expression, the densification strain, $\varepsilon_{d}$, and the nominal elasticity parameters $E_{2}$ and $E_{3}$ can be extracted, as illustrated in Figure 3b,c,d.

As shown in Figure 3b, for BCC lattices with $L_{c}=5$ mm and slender struts (η=8.66 to 2.89), the pre‐buckling regime is negligible, while the post‐buckling stiffness exhibits a scaling of $E_{2}\propto T_{b}^{4.02}$, in close agreement with the classical beam theory, which predicts $T_{b}^{4}$ dependence for bending‐dominated deformation. This strong alignment affirms that the proposed empirical model is not merely data‐driven but grounded in the underlying mechanics of lattice structures, and can capture the transition from stretching to bending‐dominated regimes. The complete data and fitted exponents are provided in Table S1 (Supporting Information) and referenced in Equations (S1)– (S3) (Supporting Information).

Furthermore, the densification strain decreases linearly with increasing beam thickness (Figure 3c), primarily due to reduced inter‐strut spacing, which accelerates contact onset during compression. During the densification phase, the nominal elastic modulus $E_{3}$ increases with beam thickness before saturating near the bulk modulus of the base material, consistent with physical expectations as the foam approaches its geometric compaction limit (Figure 3d). To further validate the model's generalizability, a BCC lattice foam with $T_{b}=0.625$ mm and $L_{c}=5$ mm was fabricated and experimentally characterized. As shown in Figure 3a, the model's prediction closely matches the experimental data, highlighting its ability to capture deformation behavior across varying geometric configurations and scales.

#### 2.1.2 Hybrid Foams

**Figure 4:**
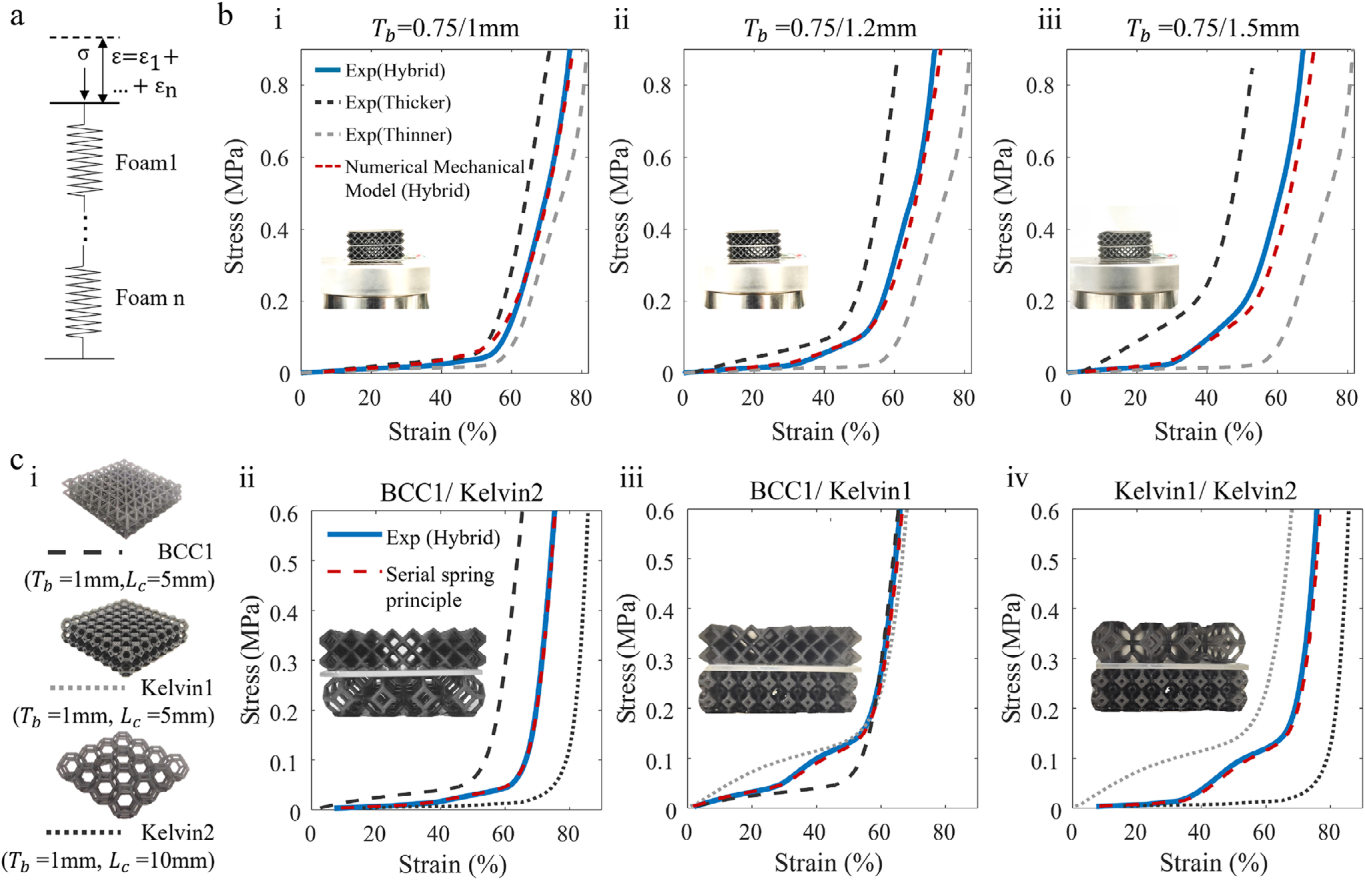
Hybrid foams with tunable mechanical properties. a) The diagram of the serial spring principle. b) The mechanical responses of hybrid foams based on BCC foams with varying beam thicknesses $T_{b}$. c) The mechanical responses of hybrid foams based on BCC and Kelvin lattices with different cell lengths $L_{c}$.

Hybrid stacked foams enable the generation of a broader range of mechanical behaviors by strategically combining two or more distinct types of lattice foam. These are laminated together in a manner that maximizes structural compatibility and performance. The resulting behavior follows the series spring principle, as illustrated in **Figure** **4**a and formalized in Equation (6).

By tuning key parameters, such as layer stiffness and densification strain ($\varepsilon_{d}$), a wide variety of non‐linear responses can be achieved. For example, stacking BCC lattice foams of varying thicknesses (Figure 4b i–iii) yields stress–strain curves with multiple linear regimes. The mechanical response of these hybrid foams can be accurately predicted by superimposing individual‐layer models, and experimental results confirm this prediction, validating both the series‐spring approximation and our mechanical framework.

BCC, Kelvin foams and other more diverse structural types with varying cell lengths can be combined to generate more intricate and diverse mechanical responses. As depicted in Figure 4c, BCC and Kelvin foams with different cell lengths can be combined to create three‐phase mechanical response characterized with nonlinear behaviors and distinct phased stiffness. As shown in Figure 4c, Using the single foam models and the series‐spring assumption, the stress‐strain behavior can be accurately predicted.

### 2.2 Hybrid Foams for Mechanical and Sensing

The SLF structures can be designed not only for mechanical properties, but also integrated with soft electrodes to develop capacitive sensors. The tuneability of the mechanical behaviors enables the force response of the sensor to be programmed.

#### 2.2.1 Capacitative Sensing of Hybrid Foams

To demonstrate the sensing capabilities of the foams and the relationship between mechanical and sensing behaviors, lattice foam sensors based on mono BCC lattice structures are fabricated with varying beam thicknesses. By laminating with top and bottom with soft conductive electrodes the foams are converted into soft sensors. Electrodes are embedded at the lamination interfaces between layers and are composed of thin, stretchable fabric interwoven with elastic and silver fibers (Figure 6a). Owing to their high compliance and minimal thickness, these electrodes exert negligible influence on the foam's mechanical behavior (less than 2% as shown in Figure 4bi– ii). As a result, the mechanical response remains largely invariant to stacking order under quasi‐static loading, while the sensor response can be effectively tuned by adjusting the sequence of the constituent layers.

**Figure 5:**
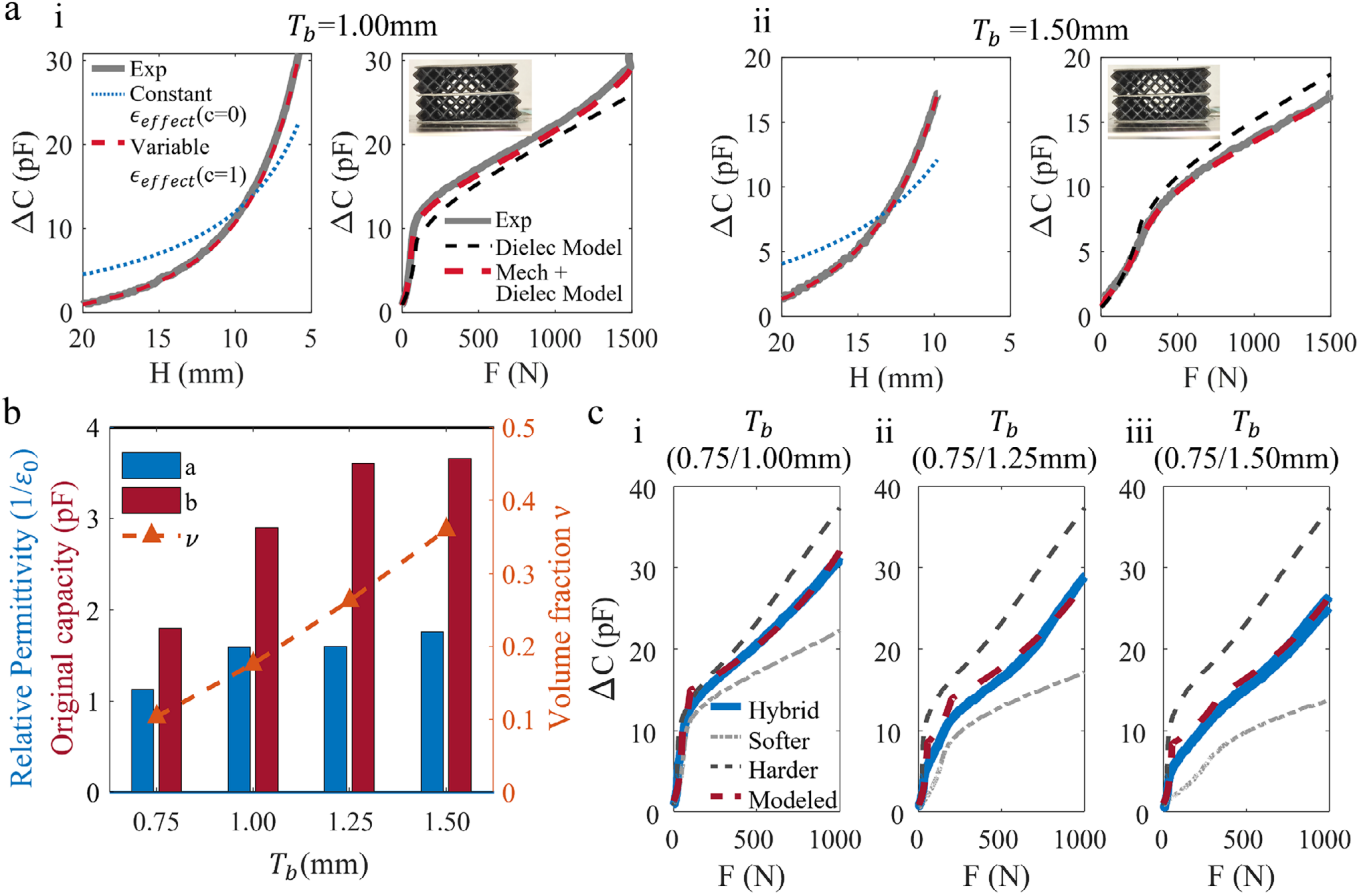
Smart hybrid foams with tunable sensing properties. a) The experimental and modeled sensing responses of soft lattice foams with varying beam thicknesses $T_{b}$. b) The relationship between dielectric parameters and beam thickness/volume fraction. c) The experimental and modeled sensing responses of smart foams based on hybrid lattice foams.

These foam sensors were characterized in terms of both mechanical and sensor performance. To build a predictive model for the sensor response, we first propose an approach based on a constant dielectric permittivity factor (**Figure** **5**a i‐ii left). However, this model does not reflect the experimental behavior, as when compressed the foam density increases, and the permittivity of the lattice foam also increases. As such, we propose a mechanics‐informed dielectric model (”mech+dielec”) model that captures this relationship between the mechanical density and the dielectric properties, as shown in Equation (4). This uses the mechanical model developed in the previous section, and leads to a more accurate model allowing the change in sensor capacitance with load to be accurately predicted. The model capturing the relationship between the mechanical density and the dielectric properties is expressed as:

(4) $$\Delta C=2*\varepsilon_{effect}A/\left(H/2\right)=2*a*{\left(H_{0}/H\right)}^{c}A/\left(H/2\right)-b$$

where ΔC is the variation in capacitance, A is the area of the sensor, $H_{0}$ and H are the origin and current height of the sensor, $\varepsilon_{effect}=a*{\left(H_{0}/H\right)}^{c}$ is the effective permittivity, a and c are dielectric factors that account for the variable permertivity of lattice foams during compression, and b is the original capacitance of the sensor. When c = 0, the permittivity of the lattice foam is assumed as constant.

By integrating BCC lattices with differing beam thicknesses into a bilayer (“hybrid”) configuration, we achieve a two‐phase capacitive response (Figure 5c). In this structure we have a “softer” layer and a “harder” layer. The softer layer lattice has thinner beams and larger cells, resulting in a lower Elastic modulus and higher sensitivity at small strains. Conversely, the “hard” layer consists of thicker beams and smaller cells, offering a higher modulus and larger sensing range. Upon compression, initially the soft layer deforms, resulting in a steep $\Delta C/C_{0}$–ε slope. As strain increases, the deformation shifts to the hard layer, thereby extending the sensing range while maintaining high sensitivity in the low‐strain regime.

#### 2.2.2 Inverse Co‐Design of a Hybrid lattice Foam Sensor

Combining different lattice foam geometries significantly expands the design space, enabling a broader spectrum of mechanical and sensory responses. To fully leverage the range of stress–strain responses, it is essential to solve the inverse design problem. Namely, to identify the geometric parameters of a foam structure that produce a specified target stress–strain curve, and the prefered sensor response.

**Figure 6:**
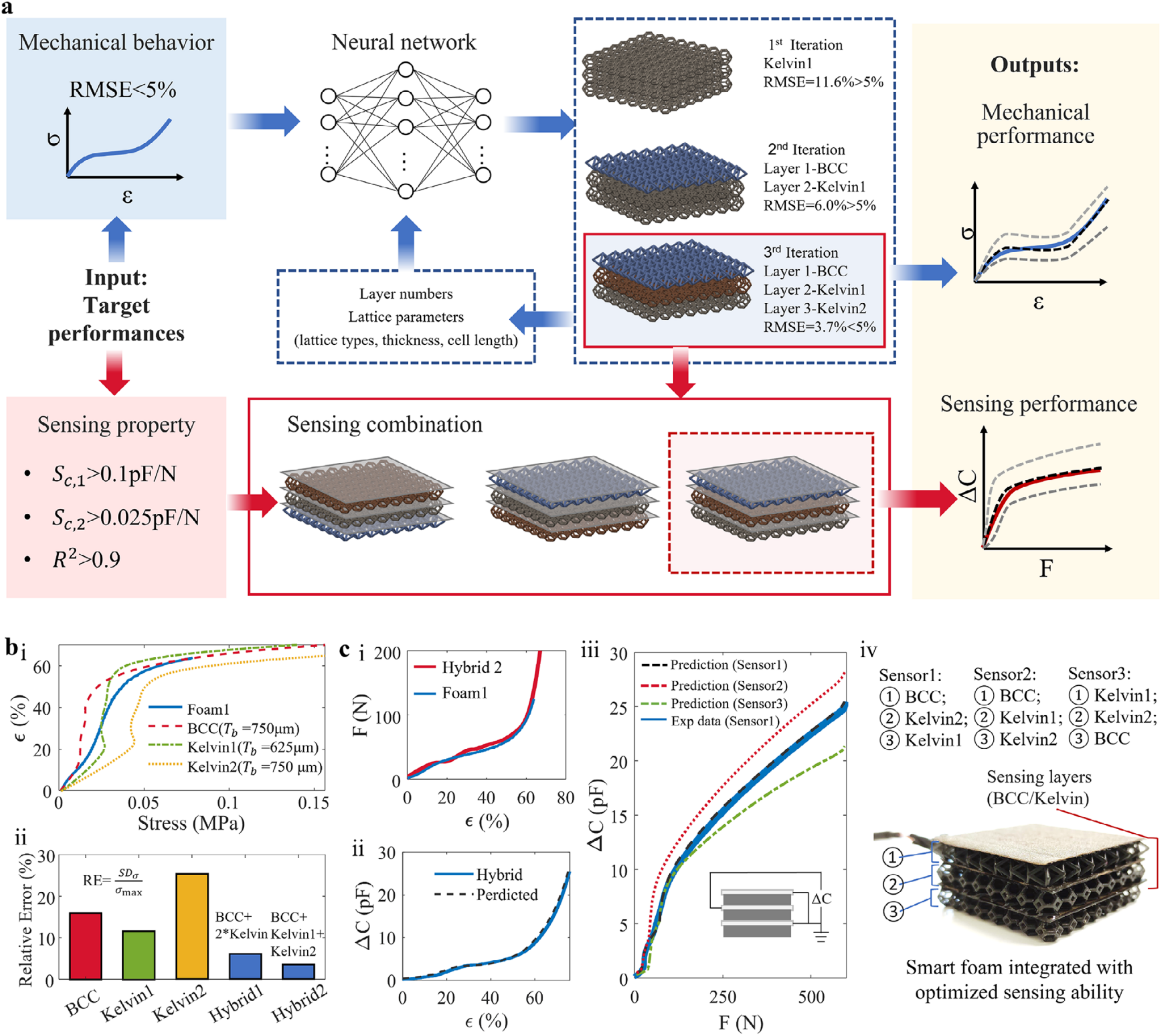
Inverse co‐design framework for achieving integrated mechanical and sensing performance. a) Schematic workflow of the inverse co‐design process based on target mechanical and sensing properties. b) Comparison of mechanical performance of mono‐material and hybrid lattice configurations, targeting the response of a reference foam (Foam1). c) The sensing response optimization of the soft lattice foam.

We address this challenge through a neural‐network based inverse design framework, which is trained on data generated from our mono‐foam mechanical models (**Figure** **6**). This neural network directly predicts the optimal geometric parameters corresponding to a desired mechanical response. This is then combined with a secondary process to optimize the layering sequence and structure to optimized the sensory performance, enabling application‐specific tuning of both mechanical and electromechanical properties.

##### Adaptive Inverse Mechanical Design

The first step of our co‐design process is the inverse design of the mechanical properties. The adaptive inverse mechanical design process is framed as a multi‐objective optimization task that aims to minimize the root‐mean‐square error (RMSE) between predicted and target stress–strain curves (Figure 6a). To ensure a balance between performance fidelity and structural simplicity, an adaptive search strategy is employed. The algorithm begins with simple mono‐material lattice candidates and incrementally expands the design space by introducing additional layers, combining different lattice types (e.g., BCC, Kelvin), or varying cell lengths'but only when the RMSE exceeds a threshold of 5%. Once a design meets the RMSE < 5% criterion, the final selection is made based on two objectives: 1) minimizing RMSE, and 2) reducing structural complexity (e.g., number of layers or transitions). This approach enables highly accurate yet practically manufacturable lattice designs.

**Figure 7:**
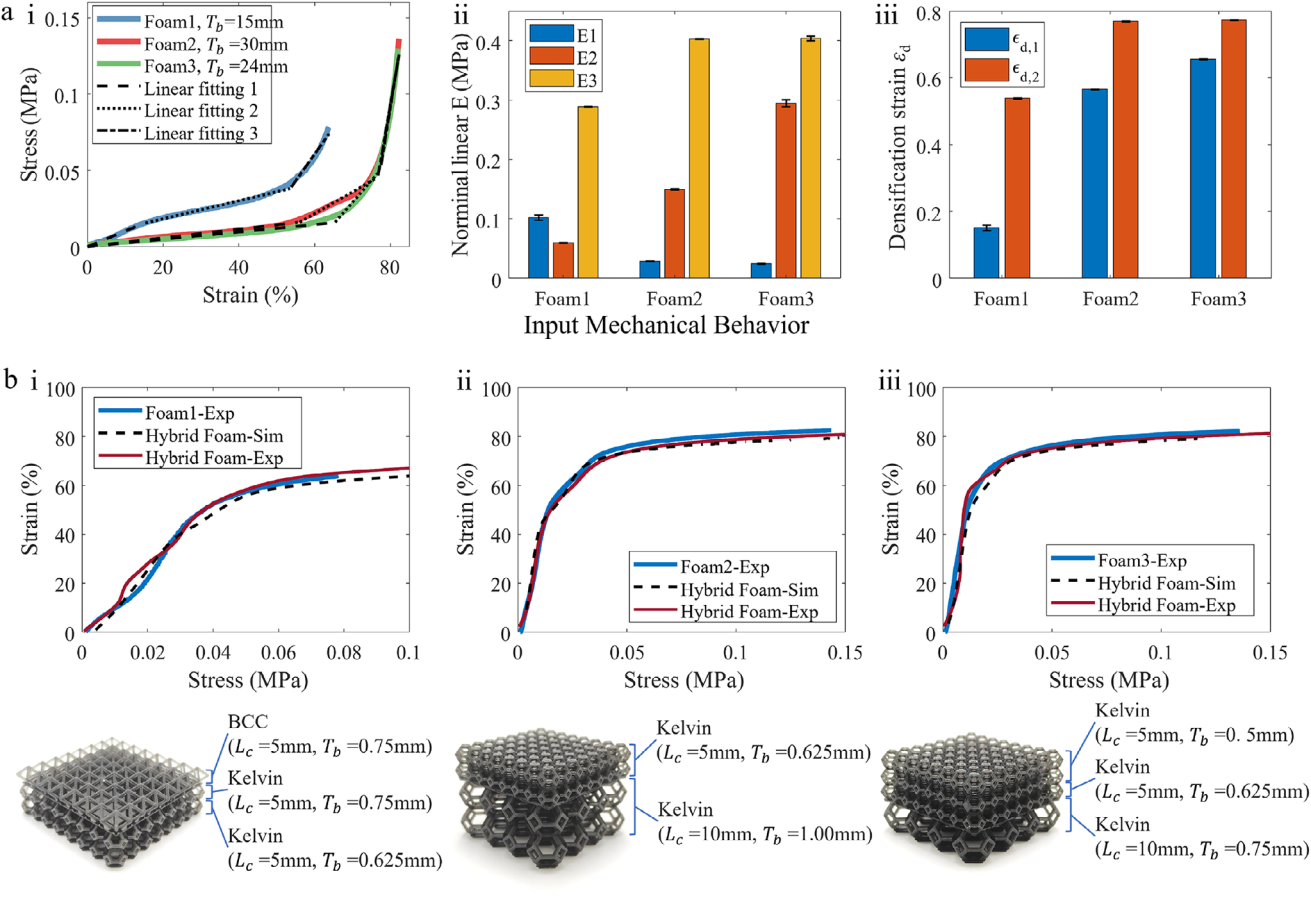
Lattice foam optimized for protective and sensing capabilities. a) The mechanical test results of three typical foams. b) The optimized results of three lattice foams with specified target mechanical behaviors.

To demonstrate the inverse design capability of the framework, we first characterized three representative 'target' foam types provided by industry experts in protective foam, Bearmind SA, which include foams best used in applications ranging from high‐impact protection to continuous load sensing, as shown in **Figure** **7**a‐i.

Their stress–strain responses were analyzed and partitioned into three characteristic regions (Figure 7a‐ii, iii), enabling quantification of effective moduli and densification strains. These curves exemplify the canonical three‐stage behavior of protective foams. Although more intricate mechanical profiles could further showcase the framework's versatility, they lie beyond the scope of our targeted real‐world applications. Notably, recent advances in inverse design for nonlinear responses^[^ ^37^, ^38^ ^]^ offer promising directions, though they are not pursued here.

To directly illustrate the different steps in the iterative and adaptive inverse‐design method, we present the foam designs for the different iterations, for the case of Foam 1 (Figures 6a and 7a). The design process began with a single‐layer Kelvin foam, which yielded an RMSE of 11.6%. To improve performance, the design space was expanded, leading to a three‐layer hybrid structure composed of BCC and Kelvin lattices. This final configuration achieved an RMSE of 3.7%, satisfying the dual objectives of mechanical fidelity and structural simplicity (Figure 6b).

The same was performed for the other two target foams, with the resulting designs shown in Figure 7b. Across all three targets, the relative deviation between the designed and target foams was approximately 3.5% (simulation) and 4.28% (experiment), as summarized in Table S2 (Supporting Information). These results validate the framework's ability to generate hybrid foam structures tailored to a wide range of mechanical specifications.

##### Co‐Design of Mechanical and Sensory Performance

The next step in our co‐design framework enables sensor optimization by exploiting the additional degree of freedom afforded by layer permutation. For instance, the Hybrid2 foam (Figure 6b‐ii) was mechanically optimized to match the response of Foam 1. However, by altering the order of the sensing layers, we achieved three distinct sensor responses' all with identical mechanical performance'demonstrated in Figure 6c(i–iii). This highlights the framework's capability for decoupled optimization of mechanical and electromechanical behavior via layer permutation. It is important to note that the observed invariance of mechanical behavior with respect to foam layer order is specific to quasi‐static conditions. Under dynamic loading, such as during impact events, this assumption may no longer hold due to potential effects from inertial mismatches, stress wave reflections, or rate‐dependent interfacial interactions between different layers.

The design of sensor 2 (based on Hybrid 2 foam, Figure 6b,c) satisfies multi‐objective performance criteria, including impact protection, wearability and sensor sensitivity and linearity, as defined in collaboration with our industry collaborator Bearmind SA (Text S1.2, Supporting Information). Specifically, the hybrid 2 foam structure achieves a densification strain of approximately 55% (Figure 6 ci), satisfying the impact absorption criterion provided by our collaborator. It demonstrates high sensitivity in both low‐ and high‐force regimes'0.11 pF/N (1–100N) and 0.050 pF/N (100–500N), each with linearity $R^{2}=0.97$. The sensor's minimum detectable force is 0.045 N (pressure resolution: 28 Pa), ensuring fine‐grained detection of both subtle and large‐scale forces. Meanwhile, the initial modulus remains 0.11 MPa (below 0.2 MPa), ensuring softness and comfort. These properties confirm that the co‐designed foam satisfies all target specifications. As such, it is selected for integration into a smart knee pad prototype. The co‐design demonstration and application‐level validation are presented in the following section.

### 2.3 Demonstration of Wearable Knee Pad with Smart Hybrid Foams

**Figure 8:**
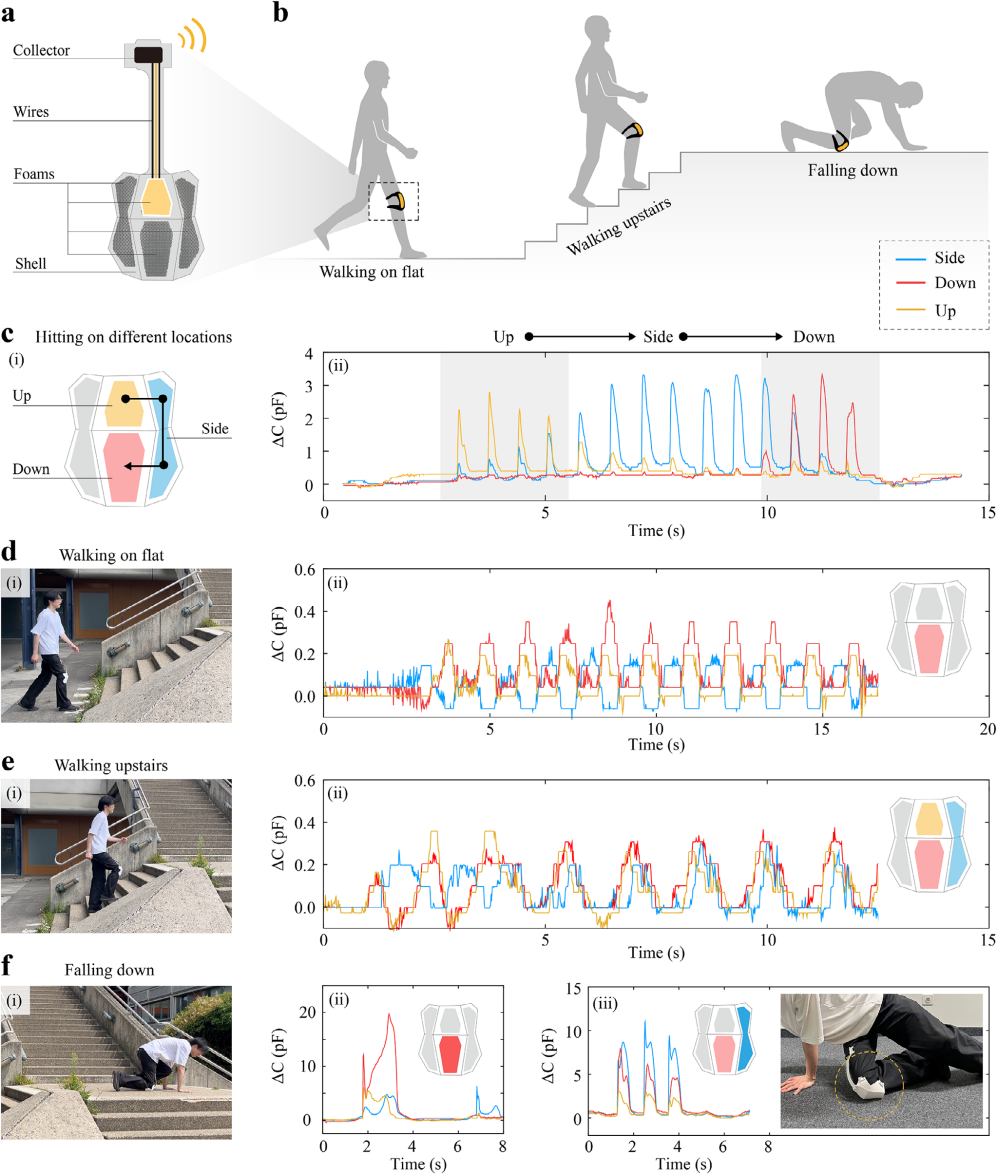
The structure and demonstrations of a smart knee pad integrated with protective and sensing capabilities. a) The structural diagram of the smart knee pad. b) Application scenarios. c) The differential sensing response to applied force in various locations. d) The sensing response during walking on flat ground. e) The sensing response during walking up stairs. f) The sensing response to falling on different areas of the knee pad.

To demonstrate the sensorized hybrid foams, we integrated the smart foam into a prototype smart knee pad designed to provide both interaction sensing and mechanical protection. The Hybrid 2 foam design was employed as the liner material within the pad. The knee pad assembly consists of three main components: an outer protective shell, multiple units of smart foam, and a signal collector (**Figure** **8**a).The distributed smart foam units allow the system not only to attenuate and detect external impacts but also to sense interaction forces exerted by the wearer.

These foam sensors are strategically positioned on the upper, lower, and lateral regions of the knee (Figure 8c‐i). As a result, the smart knee pad is capable of spatially localizing external forces based on the relative signal amplitudes from different foam segments (Figure 8c‐ii).

Additionally, it enables real‐time behavior recognition. During flat‐ground walking, for instance, the lower foam exhibits the largest signal changes, while the side foam signal dips below baseline (Figure 8d). Conversely, during stair ascent, all three foam segments show comparable signal magnitudes, with the side foam consistently above the standing‐state baseline (Figure 8e).

The system also differentiates fall types based on impact localization. In forward falls, the lower foam shows the maximum signal increase, whereas in side falls, the side foam dominates the response, though the lower foam still records substantial changes (Figure 8f). Throughout all motion scenarios, the signal remains stable and returns to baseline, indicating excellent repeatability and robustness.

This capability to detect spatial force distribution and classify user behavior has clear implications for injury prevention, gait monitoring, and behavioral analytics. It enables not only detection and mitigation of hazardous events but also detailed tracking of motion characteristics such as frequency, intensity, and duration.

## 3 Discussion

Compared to conventional embedded force‐sensing technologies in protective gear'such as accelerometers, gyroscopes, and pressure‐sensitive foams'our soft lattice foam (SLF) offers several unique advantages. Its lightweight and breathable architecture, coupled with programmable mechanical properties, enables tunable performance tailored to diverse protective requirements. Unlike traditional foams, the SLF derives both its mechanical and sensing functionality directly from the geometric configuration of the underlying lattice. This enables mechano‐electronic transduction through structural design, eliminating the need for complex surface‐mounted electronics. Furthermore, the SLF exhibits a wider range of mechanical/sensing properties owing to its reconfigurability and its ability to integrate multiple lattice architectures. Critically, our novel multi‐layer sensor structure simultaneously widens the range of possible mechanical behaviors and enables independent tuning of the sensor characteristics relative to the mechanical properties.

One of the core innovations of this work is the integration of an inverse design framework capable of generating customized SLF structures that meet target mechanical requirements while enabling selection from a range of possible sensor responses. While the forward mechanical behavior of mono‐foams can often be approximated accurately using empirical models, the inverse problem determining the optimal combination of lattice type, beam thickness, and cell length to match a desired stress–strain curve'is fundamentally more challenging. This difficulty stems from the high dimensionality of the design space and the non‐uniqueness of solutions, where multiple foam configurations can produce similar mechanical responses.

To address this, we employ a neural network (NN) to learn the nonlinear mapping from stress–strain characteristics to the corresponding set of design parameters, enabling the identification of candidate foam designs in a single step. In our framework, phenomenological models, while well suited for accurate forward prediction, are not used directly for inverse design. Instead, they generate synthetic stress–strain data across the design space, which serves as additional training input for the NN. This approach preserves the predictive strengths of the models while avoiding the high computational cost of embedding models in iterative optimization loops.

Compared with alternative optimization methods (Table S3, Supporting Information), the neural network (NN) offers several distinct advantages. Foremost, the NN is particularly well suited for inverse mapping tasks'a capability that phenomenological models alone cannot achieve. In addition, NNs are inherently adept at managing the non‐uniqueness of inverse problems, wherein multiple valid design configurations may yield similar mechanical responses. Rather than requiring exhaustive exploration of the design space, the NN converges to feasible solutions through the natural dynamics of training, thereby significantly reducing computational overhead.

Inverse design problems often involve one‐to‐many mappings, which can introduce ambiguity and instability during training. In our framework, this challenge is mitigated by the use of lattice types with clearly differentiated mechanical behaviors and by the fact that protective foams typically exhibit three distinct stress–strain stages (see Text S1.5, Supporting Information). For more complex design scenarios, ambiguity and non‐linearity can be further addressed by selecting sufficiently distinct lattice architectures, aligning the number of layers with the complexity of the target curve, or incorporating hybrid approaches that combine forward surrogate models ^[^ ^30^ ^]^ with multiple inverse networks^[^ ^37^ ^]^ or autoregressive graph‐based framework.^[^ ^38^ ^]^

A particularly important advantage of the NN‐based approach is its ability to be pre‐trained. This allows the most computationally intensive phase of the design process to be decoupled from user interaction, thereby enhancing efficiency. Once trained, the NN can be reused across multiple design tasks without retraining, in contrast to methods such as genetic algorithms (GAs), which typically require iterative optimization for each new target. As a result, the NN can generate optimal design predictions within milliseconds, enabling rapid iteration and scalability in large‐scale optimization scenarios.

The proposed approach is also highly scalable. Even within a constrained design space of only two lattice types, two cell lengths, and five beam thicknesses, there are over 3,000 possible three‐layer hybrid configurations. The NN generalizes effectively across this large space, allowing rapid identification of suitable designs. Furthermore, the inverse design framework, combined with the multi‐layer structure of the foams, enables independent tuning of the sensory response by varying the sensing layer without altering the mechanical properties. This capability is particularly powerful for co‐design in applications such as soft robotics and wearable devices. Looking ahead, the modular and generalizable nature of the NN‐based framework could be extended to co‐design foams for additional performance metrics, such as dielectric response.

Despite these advances, limitations remain. Current interfaces between lattice layers use flat interconnects, which can cause stress concentrations and localized buckling under deformation, limiting applications with highly concave or convex structures. Future work will explore smooth, topological transitions between adjacent layers, such as gradient or conformal lattice geometries, to reduce stiffness mismatches.^[^ ^39^ ^]^ Additionally, improving the interface between the foam lattice and integrated electrodes, via mechanically interlocking microstructures or surface treatments, could enhance both mechanical integrity and signal stability during repeated use.

Another avenue for future research is expanding characterization and modeling. This study focused on quasi‐static mechanical tests (0.5 mms^−1^) to provide baseline data, but dynamic testing, including drop‐weight and split‐Hopkinson pressure bar experiments, is needed to capture strain‐rate effects relevant to practical applications. Furthermore, current models assume simplified boundary conditions and regular geometries, which may not hold in complex real‐world scenarios with edge effects or non‐uniform loading. Incorporating finite element simulations and higher‐fidelity, topology‐aware modeling will improve predictive accuracy and generalizability.^[^ ^38^ ^]^

Finally, the current sensor design also has limited spatial resolution due to a single large‐area electrode pair. Incorporating high‐density, soft, stretchable electrode arrays would enable finer mapping of force distribution, while extending functionality to multimodal sensing (e.g., heart rate, temperature, bioelectric signals) could provide comprehensive data for medical monitoring or performance evaluation.

## 4 Conclusion

In summary, we introduce a modular and scalable co‐design framework that combines experimental characterization, physics informed numerical modelling, and neural network‐based inverse co‐design of mechanical and sensory properties, to enable creation of smart, sensorized soft lattice foams (SLFs). The resulting foams offer both mechanical protection and integrated capacitive sensing, widely applicable in wearable devices, soft robotics, and consumer devices. The use of a neural network enables inverse design across a highly discrete, nonlinear, and many‐to‐one design space. This allows for fast, adaptive generation of lattice geometries that match desired mechanical targets and lays the foundation for future co‐optimization with sensing and comfort functionalities. Together with advances in 3D printing, soft interfaces, and data‐driven modeling, our inverse design strategy offers a pathway toward customizable, personalizable, high‐performance wearable systems for applications in wearable devices, soft robotics and more, which could be applied to human‐centric applications including healthcare, sports tech, and elderly care.

## 5 Experimental Section

### Smart Foams Design and Fabrication

*Design and Fabrication of Soft Lattice Foams*

For rectangular lattice foams, the corresponding STL file was directly generated using OpenSCAD. When dealing with irregular shapes, the lattice array was first generated for the enclosing rectangle using OpenSCAD. Then, an intersection Boolean operation was performed between this rectangular lattice array and the CAD model of the specific shape to obtain the lattice foam with the desired geometry. The code for BCC and Kelvin lattice foam was included in the supplementary materials. To fabricate the foams, the lattice Foam is 3D printed by Halot‐Mage Pro printer (Creality 3D Technology Co. Ltd., China) with F80 elastic resin (Godsaid Technology Co. Ltd., China). The printing parameters is set in Table S4 (Supporting Information).

*Integration into Capacitative Sensors*

The electrodes were constructed using a conductive fabric woven with elastic and silver fibers. Initially, the electrodes were laser‐cut from the fabric and then connected with conductive thread to interface with the signal collector, as shown in Figure S1a‐i (Supporting Information). Subsequently, the electrodes undergo a coating process with elastic resin (F80) and were stacked with 3D‐printed lattice foams. The assembled smart foam structures cured using 400nm UV light to securely bond the electrodes and foams together, as depicted in Figure S1b (Supporting Information). Finally, the sensor was linked to the customized data aquisition device provided by Bearmind SA.

Owing to the high compliance and minimal thickness, the soft conductive electrodes exert minimal influence on the mechanical properties of the foam. To account for this small effect, a uniform correction factor was applied in the model. The experiments show a stiffness increase of less than 2.0% in most cases (Figure 4b(i–ii)); a slightly higher increase ( 3.5%, Figure 4b(iii)) was attributed to the variability of the minor fabrication. Based on this trend, a consistent 2% correction was applied to the stiffness predictions of all hybrid foam models.

### Characterization Tests and Methods

Lattice foams were tested using an Instron universal testing machine equipped with a 5 kN load cell. For smart foams integrated with capacitive sensors, the capacitance signal was recorded continuously throughout each compression. Prior to data collection, each specimen was subjected to three conditioning cycles to mitigate Mullins effects. Following this, four compression cycles were executed at a constant crosshead speed of 0.5 mms^−1^. The loading‐phase data from the four cycles were extracted and averaged to determine the foam's representative mechanical properties.

### Modelling Methods

*Numerical Mechanical Model*

The numerical model of the mechanical behavior of the foam is described by Equation (5), which captures three deformation stages'elastic, plateau, and densification'via piecewise smooth transitions. The mechanical parameters of each foam such as $E_{1}$, $E_{2}$, and $\varepsilon_{d}$ are extracted by fitting Equation (5) to experimental data. Physics‐based empirical scaling laws were then derived that link these parameters to the geometric features of the lattices (beam thickness $T_{b}$ and cell length $L_{c}$), as shown in Equations (S1), (S2), and (S3) (Supporting Information).

(5) $$\begin{array}{cc} \sigma & =E_{1}\varepsilon \;\left[1-\tanh\left(f_{s}\left(\varepsilon -\varepsilon_{b}\right)\right)\right]/2\\ & \;+\left(E_{2}\left(\varepsilon -\varepsilon_{b}\right)+E_{1}\varepsilon_{b}\right)\;\left[\tanh\left(f_{s}\left(\varepsilon -\varepsilon_{b}\right)\right)-\tanh\left(f_{s}\left(\varepsilon -\varepsilon_{d}\right)\right)\right]/2\\ & \;+\left(E_{3}{\left(\varepsilon -\varepsilon_{d}\right)}^{3}+E_{2}\left(\varepsilon -\varepsilon_{b}\right)+E_{1}\varepsilon_{b}\right)\;\left[1+\tanh\left(f_{s}\left(\varepsilon -\varepsilon_{d}\right)\right)\right]/2 \end{array}$$

here, $f_{s}=30$ is a smoothing factor, and the transition points $\varepsilon_{b}$ and $\varepsilon_{d}$ denote the buckling and densification strains, respectively.

Then, the stress–strain response of hybrid foams was synthesized using a serial spring model that sums inverse stiffness contributions of layered mono‐foams:

(6) $$\begin{array}{cc} \sigma_{1} & =f_{1}\left(\varepsilon \right)=f\left(\varepsilon ,P_{g,1}\right),\;\sigma_{2}=f_{2}\left(\varepsilon \right)=f\left(\varepsilon ,P_{g,2}\right)\\ \varepsilon_{hb} & =g_{hb}\left(\sigma \right)=g_{1}\left(\sigma \right)+g_{2}\left(\sigma \right),\;\text{where}\;g_{i}\left(\sigma \right)=\left(1+\lambda_{e}\right)f_{i}^{-1}\left(\sigma \right) \end{array}$$

here, f represents the numerical stress–strain function for mono‐foams, and $\lambda_{e}=2\%$ accounts for integration artifacts due to embedded electrodes. This model allows rapid simulation of arbitrary hybrid combinations based on unit‐cell geometries.

*Numerical Dielectrical Model*

The model capturing the relationship between the mechanical density and the dielectric properties was expressed as Equation (4). By fitting the dielectrical response, the parameter of Equation (4) can be obtained for each foam, which could be utilized to predict the sensing behavior of smart foams based on their measured or modelled mechanical behaviors. Furthermore, the Equation (4) could be normalized and rewritten by replacing the electrode distance H with strain ε as below:

(7) $$\begin{array}{c} \Delta C=2*\varepsilon_{effect}A/\left(\varepsilon H_{0}/2\right)-C_{0}=2*a*{\left(1/\varepsilon \right)}^{c}A/\left(\varepsilon H_{0}/2\right)-C_{0} \end{array}$$

Considering the same lattice material but with varying thicknesses denoted as $H_{0,1}$, the permittivity factor remains constant. Consequently, the original capacitance of a new foam with varied thickness can be expressed as $C_{0,1}=\frac{H_{0,1}C_{0}}{H_{0}}$. Here, the electrode area A is treated as constant based on the assumption of uniaxial compression, the foam's low thickness‐to‐area ratio, and its high compressibility. Empirical testing up to 60% strain showed negligible lateral expansion, justifying this simplification within the experimental conditions. However, it was noted that under free‐form deformation or alternate boundary conditions, A may become strain‐dependent. This limitation was acknowledged, and future models may incorporate lateral compliance effects where relevant.

### Adaptive Inverse Mechanical Design

*Primary Objective*. The goal of the inverse co‐design framework was to minimize the root‐mean‐square error (RMSE) between the predicted and target stress–strain curves. A candidate design is accepted when RMSE < 5%. The RMSE is computed as:

(8) $$\mathrm{RMSE}=\sqrt{\frac{1}{N}\sum_{i=1}^{N}{\left(\varepsilon_{\mathrm{target}}\left(\sigma_{i}\right)-\varepsilon_{\mathrm{proposed}}\left(\sigma_{i}\right)\right)}^{2}}$$

*Adaptive Solution Generation Strategy*. To identify suitable designs, the neural network adopted a progressive search strategy that added design complexity only when necessary (see more details in Figure S2 and Text S1.3, Supporting Information):

- **Step 1: Initial Proposals**
  Begin with simple single‐layer mono‐lattice structures (e.g., all‐Kelvin or all‐BCC) to assess feasibility.
- **Step 2: Design Space Expansion**
  If RMSE>5%, progressively expand the design space:
  - Increase the number of layers (from 1 to 2/3)
  - Combine different lattice types (e.g., BCC + Kelvin)
  - Vary cell lengths ($L_{c}$ = 5–10 mm)
- **Step 3: Final Selection**
  Among all candidate solutions that meet the RMSE threshold, the framework selects the one with:
  - The least design complexity (e.g., minimal number of layers)
  - The lowest RMSE

### Neural Network Design For Hybrid Foam Inverse Design

*Data Generation and Preprocessing*

First, the experiment data of three foam types and the generated modeling data from the model were collect and normalized. For each type of lattice foam, five groups experimental data and five groups modeling data with different beam thickness were invovled. Then, to simulate the mechanical behavior of hybrid foam combinations, a combinatorial approach was employed. From a training set of 30 distinct foam samples, all possible ordered combinations of three foams were generated using nested loops, resulting in a total of 303=4060 unique hybrid configurations. For each combination: The input features were computed as the mean strain response across the selected foams. The target outputs were the indices of the selected foams, representing the hybrid composition. Let:

(9) $$\begin{array}{cc} & X\in R^{30\times N}\;\text{(strain features)},\\ & Y\in R^{6\times N},\;Y=\left[\begin{array}{c} Y_{1}\\ Y_{2} \end{array}\right]\;\text{(foam indices)} \end{array}$$

where N denotes the number of hybrid combinations; $Y_{1}\in \left\{1,2,3\right\}$ specifies the foam type index for three discrete foam categories; and $Y_{2}\in \left[0.5,\;1.5\right]$ represents the beam thickness index. The training data were normalized and ordered according to the foam type numbers and layer thickness, thereby improving data continuity. More details see in Text S1.5 (Supporting Information).

*Mitigation of One‐to‐Many Mapping Problems*

The one‐to‐many mapping issue also presented challenges in inverse design, as it could lead to instability, training difficulties, and evaluation ambiguity in neural networks. In this study, this risk was minimized because the three selected lattice foam types exhibit distinctly different mechanical behaviors, and commercial protective foams typically display only three major stress–strain stages. As a result, the three‐layer designs did not experience severe mapping ambiguity. For more complex cases, the risk could be mitigated by matching the target curve's complexity with an appropriate number of layers and selecting lattice types that were clearly distinct across the performance range. In addition, advanced strategies'such as combining separate forward surrogate networks with multiple independently trained inverse networks, as demonstrated by Ha et al.^[^ ^37^ ^]^'can further reduce the impact of one‐to‐many mapping.

### The Knee Pad Design Based on SLF

The shell of the knee pad was 3D‐printed using an FDM printer and TPU filament (Flex Medium, Extrudr). The smart foam consisted of two layers of Kelvin lattice and one layer of BCC lattice foam, with a beam thickness of 0.65, 0.75, and 0.75mm, respectively. The signal collector was connected to the knee pad using a TPU band embedded with electrical wires, allowing it to be securely fastened outside the knee area. Finally, the knee pad and signal connector are affixed to the wearer's leg using elastic straps.

### Statistical Analysis

All statistical analyses were performed using OriginPro 2023 (OriginLab Corporation) and MATLAB R2023a (MathWorks Inc.), with built‐in toolboxes used for curve fitting, regression analysis. All quantitative data were presented as the mean ± standard deviation (mean ± SD). Mechanical properties (e.g., modulus, plateau stress, densification strain) and sensor characteristics (e.g., sensitivity, linearity) were computed by averaging the loading phase curves of the four final cycles.

## Conflict of Interest

The authors declare no conflict of interest.

## Supporting information

Supporting Information

Supplemental Video 1

Supplemental Video 2

## Data Availability

The data that support the findings of this study are available in the supplementary material of this article.
