# Supplementary material for: Inverse Co‐Design of Mechanical And Sensory Properties in Soft Lattice Foams for Multifunctional Wearables
Source: Adv Sci (Weinh). 2025 Sep 23;12(44):e07102. doi: 10.1002/advs.202507102 (PMC12667523; doi:10.1002/advs.202507102)
Supplement: Supplementary file 1 — Supporting Information [file ADVS-12-e07102-s003.pdf]

Supplementary Materials

Supplementary Material List

S1 Supplementary Text

S1.1 Modeling parameters of Kelvin and BCC Lattice Foams
S1.2 Target Foam Property for knee pad design
S1.3 Example: Adaptive Mechanical Optimization for 'Foam 1'
S1.4 Workflow of the Inverse Co-Design of Soft Lattice Foams
S1.5 Neural Network Design for Hybrid Foam Inverse Design

S2 Supplementary Tables

Table S1: Scaling exponents of modulus vs. slenderness ratio across pre- and post-buckling regimes for different lattice cells.
Table S2: Inverse design results of mechanical behaviors
Table S3:Comparison of inverse design strategies for soft lattice foam generation.
Table S4: 3D printing parameters of the soft lattice foam

S3 Supplementary Figures

Figure S1: The fabrication of the soft lattice foam.
Figure S2: Diagram of Hybrid lattice foam design and fabrication workflow.
Figure S3: Stress-strain curves for BCC and Kelvin lattice foams under different loading rates.

S4 Supplementary Videos

Video S1: Hammer hitting sensing and localization.
Video S2: Behavior tracking of flat walking, stair climbing and falling.

S1 Supplementary Text

S1.1 Modeling parameters of Kelvin and BCC Lattice Foams

Kelvin Lattice ($L_c = 5\,\mathbf{mm}$): This structure has a moderate slenderness ratio ($\eta = L_b/T_b = 3.54$ to 1.18). The fitted exponents show that pre-buckling deformation is mainly stretching-dominated, while post-buckling response is influenced increasingly by bending:

$$\begin{aligned} &E_1 = 0.42 \cdot T_b^{2.66}, \quad E_2 = 0.258 \cdot T_b^{3.10} - 0.018, \quad E_3 = 125.0 \cdot T_b^{1.84} - 1.76 \\ &\varepsilon_b = 0.367 \cdot T_b + 0.166, \quad \varepsilon_d = -0.21 \cdot T_b + 0.752 \end{aligned} \tag{S1}$$

The exponent $n = 2.66$ in E_1 aligns with stretching-dominated beam theory (ideal $n = 2$), with slight elevation due to shear and in-plane effects. Post-buckling behavior transitions into partial bending with $n = 3.10$.

Kelvin Lattice ($L_c = 10\,\mathbf{mm}$): This version exhibits higher slenderness ratios ($\eta = 7.07$ to 2.36), causing early onset of buckling and dominant bending deformation in both regimes:

$$\begin{aligned} &E_1 = 0.0256 \cdot T_b^{3.63}, \quad E_2 = 0.0116 \cdot T_b^{3.87} + 0.005, \quad E_3 = 141.6 \cdot T_b^3 - 379.9 \cdot T_b^2 + 219.7 \cdot T_b + 55.32 \\ &\varepsilon_b = 0.15, \quad \varepsilon_d = -0.311 \cdot T_b + 0.968 \end{aligned} \tag{S2}$$

The elevated E_1 exponent of 3.63 shows that even pre-buckling deformation is largely bending-dominated due to long, slender struts. Post-buckling E_2 approaches the ideal bending exponent ($n = 4$).

BCC Lattice ($L_c = 5\,\mathbf{mm}$): For this lattice, the pre-buckling phase is negligible. The post-buckling regime dominates and is highly consistent with pure bending theory:

$$\begin{aligned} &E_2 = 0.0884 \cdot T_b^{4.02} + 0.0115, \quad E_3 = \frac{45.6}{1 + e^{-8.8 \cdot T_b + 5.2}} - 0.24 \\ &\varepsilon_d = -0.29 \cdot T_b + 0.79 \end{aligned} \tag{S3}$$

The fitted exponent $E_2 \propto T_b^{4.02}$ strongly validates the bending-dominated nature of BCC lattices, especially given their high η range of 2.89–8.66.

S1.2 Target Foam Property for knee pad design

The knee pad application utilizes the hybrid foam labeled 'Foam 1' in Figure 6a, co-designed in collaboration with Bearmind SA to meet industry-specific criteria for protective sports equipment. The optimization targeted three critical objectives:

- **Impact Protection:** The foam was engineered to exceed a densification threshold of 45% strain to avoid bottoming-out during impact.
- **Sensing Precision and Range:** To support gait and impact monitoring, the target sensor response was:
  - Sensitivity ≥ 0.10 pF/N in the 1–100 N range,
  - Sensitivity ≥ 0.025 pF/N in the 100–500 N range,
  - Linearity $R^2 > 0.95$ across both regimes.
- **Wearability:** For user comfort and compliance, the initial modulus was constrained to ≤ 0.2 MPa.

S1.3 Example: Adaptive Mechanical Optimization for 'Foam 1'

- **Initial Design:**
  Single-layer mono-foam using Kelvin ($T_b = 6.25$ mm, $L_c = 5$ mm) $\rightarrow$ RMSE = 11.6%
- **First Expansion:**
  Two-layer hybrid using Kelvin foams:
  - Layer 1: $T_b = 0.75$ mm, $L_c = 5$ mm
  - Layer 2: $T_b = 1.25$ mm, $L_c = 10$ mm

  $\rightarrow$ RMSE = 6.1%
- **Final Design:**
  Three-layer hybrid combining BCC and Kelvin:
  - Layer 1: BCC ($T_b = 0.65$ mm, $L_c = 5$ mm)
  - Layer 2: Kelvin ($T_b = 0.75$ mm, $L_c = 5$ mm)
  - Layer 3: Kelvin ($T_b = 1.25$ mm, $L_c = 5$ mm)

  $\rightarrow$ RMSE = **3.7%**—successfully meets the target.

S1.4 Workflow of the Inverse Co-Design of Soft Lattice Foams

The inverse co-design framework integrates both mechanical and sensing performance into the design of hybrid soft lattice foams. The workflow is illustrated in Figure S2 and consists of the following key stages:

Step 1: Mono-Foam Characterization. A library of mono-lattice foams is first fabricated using various lattice types (e.g., Kelvin, BCC), beam thicknesses (T_b), and cell lengths (L_c). Each configuration is experimentally characterized to extract its stress–strain response. These data are used to construct physics-informed mechanical models for each mono-foam.

Step 2: Mechanics Database Construction. Using the experimentally validated mono-foam models, a database of potential hybrid foam responses is generated based on serial spring hybridization principles. This includes different layer permutations, lattice types, and geometric parameters.

Step 3: Neural Network Training. A feedforward neural network (three hidden layers with 33, 66, and 33 neurons) is trained on the hybrid foam database to learn the mapping between mechanical responses and foam design parameters. The network is then used to predict a candidate design that matches a target stress–strain curve.

Step 4: Inverse Mechanical Design via RMSE Minimization. The network's proposed design is evaluated using a numerical forward model. The root-mean-square error (RMSE) between the predicted and target stress–strain curves is computed with Eq. 7. If RMSE $< 5\%$, the solution is accepted. Otherwise, the design space is expanded—by increasing the number of layers, introducing new lattice geometries, or varying cell lengths—and the network is retrained or queried again.

Step 5: Sensing Optimization. Once the mechanical requirements are satisfied, the dielectric model is employed to evaluate the sensing response for all valid layer permutations. The configuration with the closest match to the desired sensing behavior (e.g., sensitivity, range) is selected.

Step 6: Fabrication of Sensorized Hybrid Foam. The final selected hybrid foam structure is fabricated with embedded electrodes and dielectric elastomer layers to realize a fully integrated smart foam (see Figure S1). This device is used in downstream applications such as wearable protection (e.g., smart knee pad).

S1.5 Neural Network Design for Hybrid Foam Inverse Design

Neural Network Architecture A feedforward neural network (FNN) was constructed using MATLAB's `feedforwardnet` function. The architecture consists of:

- **Input layer**: 30 neurons (strain feature vector dimension)
- **Three hidden layers**:
  - Layer 1: 35 neurons
  - Layer 2: 71 neurons
  - Layer 3: 33 neurons
- **Output layer**: 6 neurons (predicting foam indices)

Training Procedure The network was trained using supervised learning with the following setup:

- **Training function**: Default backpropagation (`train`)
- **Loss function**: Mean squared error (MSE)
- **Training data**: All generated hybrid strain combinations and corresponding foam indices. 90% data is used for training and 10% data is used for validation.

Model Evaluation The trained model was evaluated on the training data to assess prediction accuracy.

- For each prediction, the root mean square error (RMSE) between the predicted and actual foam indices was required to be less than 2.5%.
- Overall performance was evaluated based on two criteria: (i) the proportion of validation samples with an error greater than 1% should remain below 10%, and (ii) the global RMSE across all validation samples should be less than 2.5%.

Generalization Test To assess the generalization capability of the trained network, the strain response of a commercial foam sample was used as input. The network predicted the corresponding foam indices based on this response. Subsequently, the modeled output foam design was compared against the original commercial foam through both simulation and experimental validation.
Finally, the trained network was saved for future deployment using.

S2 Supplementary Tables

Supplementary Table S1 Scaling exponents of modulus vs. slenderness ratio across pre- and post-buckling regimes for different lattice cells.

lattice Type	Stretching-dominated (Ideal)	Kelvin ($L_c = 5$ mm)	Kelvin ($L_c = 10$ mm)	BCC ($L_c = 5$ mm)	Bending-dominated (Ideal)
Slenderness Ratio \ $\eta = L_b/T_b$	-	1.18 – 3.54	2.36 – 7.07	2.89 – 8.66	-
Pre-buckling Exponent n (E_1)	2	2.66	3.63		4
Post-buckling Exponent n (E_2)	2	3.10	3.87	4.02	4

Supplementary Table S2 Inverse design results of mechanical behaviors

Percentage Error from Target Foam	Foam 1	Foam 2	Foam 3	Average
simulation result (%)	2.74	3.61	4.21	3.52
experiment result (%)	3.71	4.79	4.34	4.28

Supplementary Table S3 Comparison of inverse design strategies for soft lattice foam generation.

**Criterion**	**Neural Network (NN)**	**Genetic Algorithm (GA)**	**Brute-force Search**
RMSE (Target Fit)	< 5%	~5–6%	< 5%
Average Output Time	< 1 second (Pre-trained)	1–5 minutes	> 30 minutes
Handles Non-uniqueness	Can Learn distribution of valid solutions with multiple independent Neural networks	Can explore disconnected solution regions with populations of solutions	Requires exhaustive enumeration
Ideal for massive or interactive design	Ideal choice (can generate designs instantly once trained)	Population-based evolution supports interaction, but speed decreases significantly for large-scale tasks	Impractical for massive and interactive design (Exhaustive enumeration makes it too slow for practical use)
Scalability to 3-5 layers (Reconstruction difficulty and computational load increase)	Good	Limited	Poor
Requires Retuning per Target	None	Requires re-evolution or tuning for every target	No need to retrain
Integratable with Future Tasks	Easy	Medium	Hard

Supplementary Table S4 3D printing parameters of the soft lattice foam

Layer height (mm)	0.1	Light-off Delay (s)	8
Bottom layer Count	4	Lifting Distance (mm)	13
Exposure Time (s)	5	Lifting Speed (mm/min)	90
Bottom Exposure Time (s)	55		

S3 Supplementary Figures

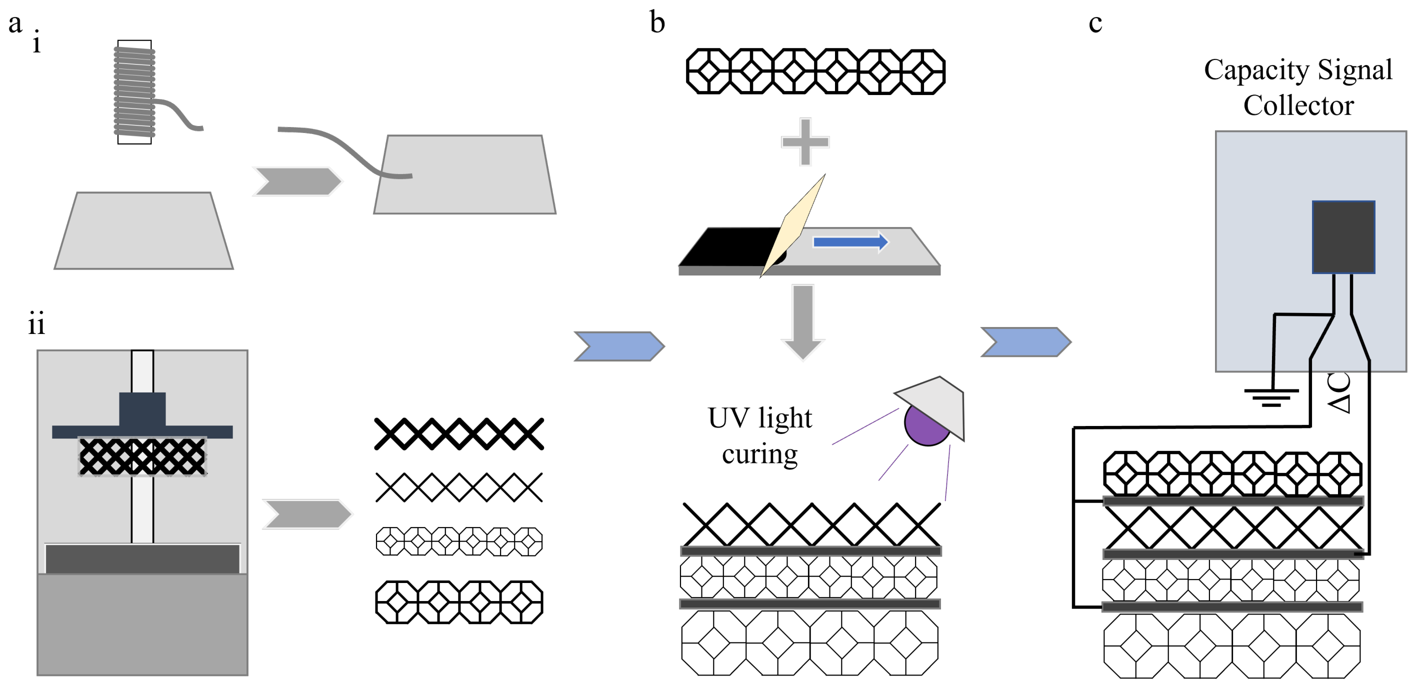

Supplementary Figure S1 The fabrication of the soft lattice foam. a) The preparation of the conductive fabric layers and mono lattice foam layers. b) The assembly of the lattice foams and conductive layers. c) Connecting the smart foam to the capacitance meter.

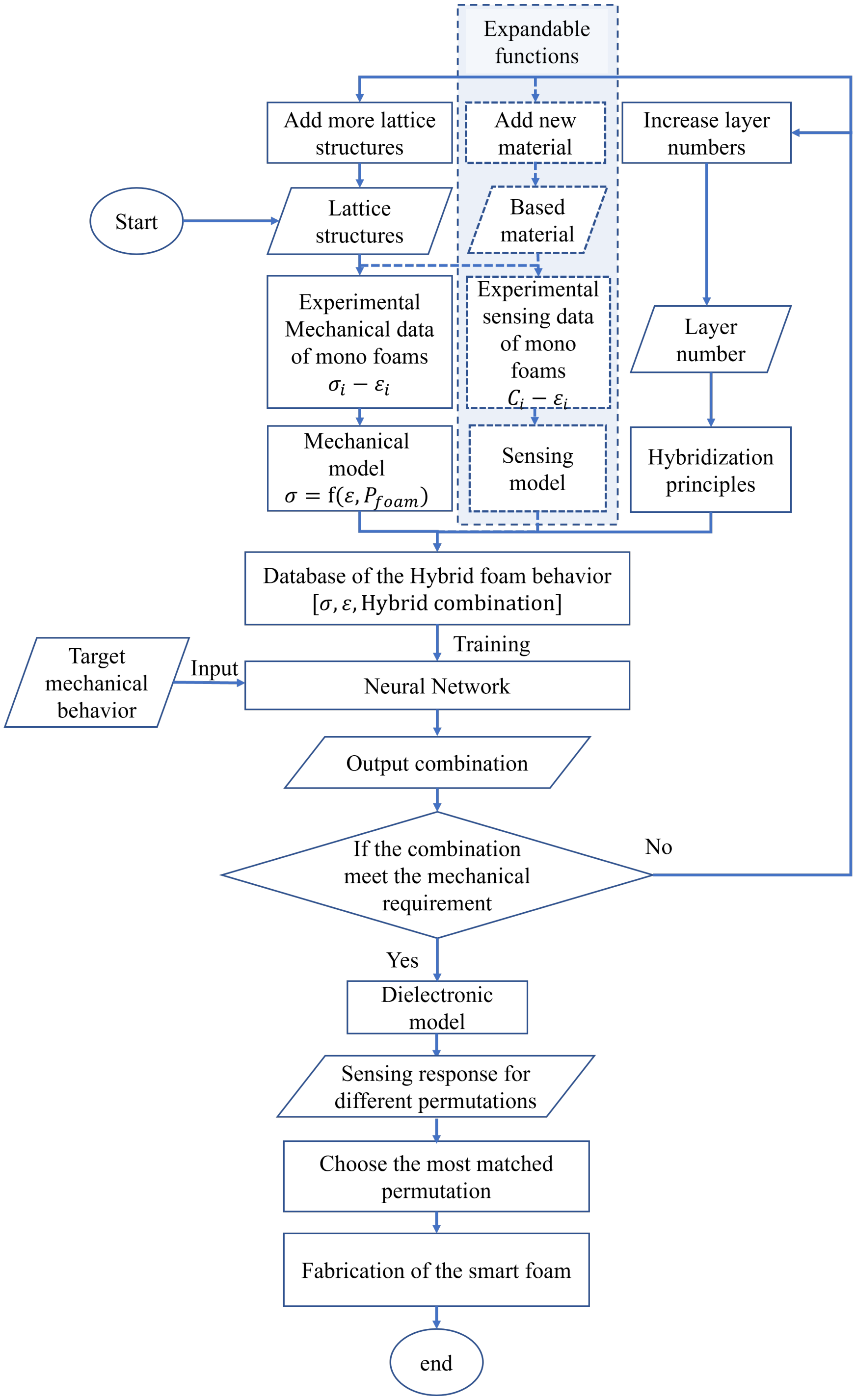

Supplementary Figure S2 Diagram of Hybrid lattice foam design and fabrication workflow.

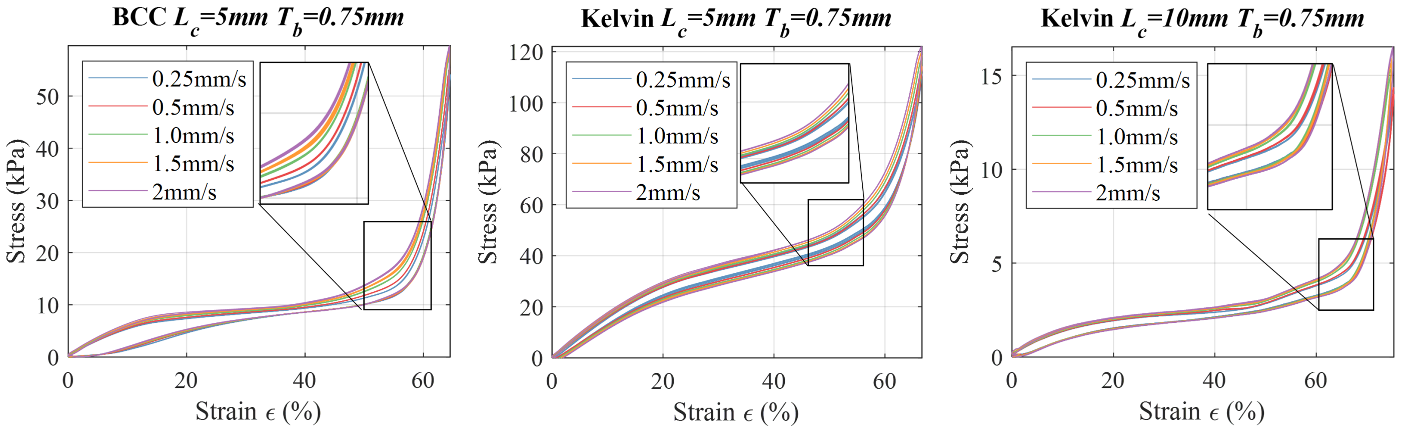

Supplementary Figure S3 Stress-strain curves for BCC and Kelvin lattice foams under different loading rates. The curves illustrate the mechanical response at varying strain rates: 0.25 mm/s, 0.5 mm/s, 1.0 mm/s, 1.5 mm/s, and 2 mm/s.
